# Variation in Taxonomic Composition of the Fecal Microbiota in an Inbred Mouse Strain across Individuals and Time

**DOI:** 10.1371/journal.pone.0142825

**Published:** 2015-11-13

**Authors:** Yana Emmy Hoy, Elisabeth M. Bik, Trevor D. Lawley, Susan P. Holmes, Denise M. Monack, Julie A. Theriot, David A. Relman

**Affiliations:** 1 Department of Microbiology and Immunology, Stanford University School of Medicine, Stanford, California, United States of America; 2 Department of Statistics, Stanford University, Stanford, California, United States of America; 3 Department of Biochemistry, Stanford University School of Medicine, Stanford, California, United States of America; 4 Howard Hughes Medical Institute, Stanford University, Stanford, California, United States of America; 5 Department of Medicine, Stanford University School of Medicine, Stanford, California, United States of America; 6 Veterans Affairs Palo Alto Health Care System, Palo Alto, California, United States of America; Columbia University, UNITED STATES

## Abstract

Genetics, diet, and other environmental exposures are thought to be major factors in the development and composition of the intestinal microbiota of animals. However, the relative contributions of these factors in adult animals, as well as variation with time in a variety of important settings, are still not fully understood. We studied a population of inbred, female mice fed the same diet and housed under the same conditions. We collected fecal samples from 46 individual mice over two weeks, sampling four of these mice for periods as long as 236 days for a total of 190 samples, and determined the phylogenetic composition of their microbial communities after analyzing 1,849,990 high-quality pyrosequencing reads of the 16S rRNA gene V3 region. Even under these controlled conditions, we found significant inter-individual variation in community composition, as well as variation within an individual over time, including increases in alpha diversity during the first 2 months of co-habitation. Some variation was explained by mouse membership in different cage and vendor shipment groups. The differences among individual mice from the same shipment group and cage were still significant. Overall, we found that 23% of the variation in intestinal microbiota composition was explained by changes within the fecal microbiota of a mouse over time, 12% was explained by persistent differences among individual mice, 14% by cage, and 18% by shipment group. Our findings suggest that the microbiota of controlled populations of inbred laboratory animals may not be as uniform as previously thought, that animal rearing and handling may account for some variation, and that as yet unidentified factors may explain additional components of variation in the composition of the microbiota within populations and individuals over time. These findings have implications for the design and interpretation of experiments involving laboratory animals.

## Introduction

The intestinal microbiota plays a number of important roles in animal health, including gut development, extraction of energy from food, protection against pathogens, and development, maturation, and responsiveness of the immune system [1,2]. Alterations in the composition of the intestinal bacterial communities have been implicated in obesity, inflammatory bowel disease, diabetes, and a variety of disease states [2–4]. However, a more detailed understanding of the range of microbiota compositional states during health would help in efforts to define and characterize disease-associated communities.

In humans, there are significant individual-to-individual differences in the phylogenetic composition of the indigenous microbiota. These differences are thought to reflect host genetics and environmental exposures, such as diet [5,6], but the relative contributions of genetics and environment remain poorly characterized. Comparisons of twins have yielded conflicting results regarding the degree of similarity in microbiota phylogenetic composition between monozygotic and dizygotic twin pairs and the magnitude of the effect of genetics [5,7–9].

Laboratory animals provide a more controlled setting in which to examine the relationship between host genetics, diet, other environmental factors and composition of the microbiota [10]. Studies comparing the microbiota of mice have shown greater differences among the microbiota of laboratory mice of different strains than among different mice of the same strain [10–20]. However, since there is a strong litter effect (mice have a more similar microbiota to that of their mother than to that of unrelated mice) [21–24], some of the strain-associated differences might be due to the fact that different strains have been bred separately for many generations. Studies of host quantitative trait loci (QTL) in mice identified QTLs linked to the relative abundances of specific microbial taxa, arguing for a role of host genotype in determining microbiota composition [22,25]. Other studies using linkage analysis, investigating the effect of specific genes on the microbiota, or comparing related and unrelated lineages within a mouse strain have also found links between genetics and the composition of the microbiota [10,26–30].

In addition to genetics, environmental factors and stochastic effects have been shown to affect the composition of the microbiota. Within inbred mouse strains, inter-individual variation of the microbiota has been reported [10,31,32]. Despite a strong litter effect, in which genetic relatedness is expected to play a major role, there are measurable differences in the composition of the intestinal microbiota among littermates [21,23]. In models involving the simplified altered Schaedler flora [33–36], cohabitation at the time of weaning had a greater effect on the relatedness of microbiota among mice than co-membership in the same litter of origin [20,37], which may be due to the stabilization of the microbiota after weaning [38]. This suggests that in addition to genetics and the initial maternally-derived inoculum, later events can impact the composition of the microbiota. Diet is one such factor that has been shown to have a large impact on the composition of the microbiota [25,39–41]. Changes in fat or carbohydrate content induce shifts in the abundance of taxa over short timescales [6,24,40–44], and long-term diet preferences are also associated with patterns of microbiota composition [6]. Cohabitation has also been shown to affect the microbiota, [10,15,20] and components of the microbiota can be transferred between cohoused individuals [29]. The effect of cohabitation–or in the case of mice, cage effect–can result in differences in responses to perturbations [45,46]. Differences in the microbiota between mice of the same strain from different vendors [16,31] and between mice housed in different rooms of the same facility [46,47] have been reported. That may be the result of a combination of genetics, environment at the point of weaning, and cohabitation as adults.

In studies of adult laboratory mice in which diet, medication, and general environmental conditions are controlled, the composition of the intestinal microbiota has been reported to be relatively stable [38,48,49]. However, some of these studies relied on denaturing gradient gel electrophoresis (DGGE), which does not provide a high resolution picture of microbial community composition, or they relied on mice with intestinal communities of reduced-complexity, such as altered Schaedler flora [48,49]. Despite these limitations, a comparative analysis of intestinal community composition from 19 different laboratory mice based on DGGE showed significant differences in the community composition of individual mice over the course of a few weeks [50], and analysis of fecal metabolites from laboratory mice found variation over time [32] suggesting that time is a potentially important source of variation of the composition of microbiota in laboratory animals.

The aim of this study was to characterize variation in the phylogenetic composition of the fecal microbiota of a laboratory mouse strain during states of health. We examined the fecal microbiota between and within individuals over time in genetically identical, inbred female mice housed under the same environmental conditions. Among our findings, we show that time and vendor shipment group (pool-weaning group) membership greatly affect the composition of the microbiota of an individual.

## Materials and Methods

### Animals

Female 129X1/SvJ mice were purchased from Jackson Laboratories (Bar Harbor, ME), and were five to eight weeks of age at the time of shipment to our laboratory. They had been pool-weaned at 3 weeks (+/- 3 days) of age and had remained in the same pool until shipment. We attempted to obtain additional information from Jackson Laboratories on litter membership of these mice, but that information was not available. Mice were housed in the Stanford University School of Medicine animal facility for one to two weeks before the beginning of each experiment, as described by Lawley *et al*. [51]. Mice were maintained in specific pathogen free conditions and were given food of a single type and from a single source (ProLab 3000 RMH; Purina Mills, Inc., St. Louis, MO), as well as reverse osmosis-filtered water *ad libitum*. Food, bedding, and water were changed every seven days. All mice were housed in the same room in filter top cages, with three to five mice per cage. Mice were marked so that individual mice could be followed for the duration of the experiments. Mice were numbered by shipment group (I-IV), cage (A-K), and individual (1–46), resulting in a three part identification code, e.g., “I_A_1”. All animal experiments were performed in accordance with the recommendations and approval of the Stanford University Institutional Animal Care and Use Committee.

### Sample collection

Fecal samples were collected from 46 individual female mice in eleven separate cages. One to four samples were collected from each mouse over a period of no greater than two weeks. In addition, 20–23 fecal samples were collected from each of four mice over a 218–236 day period. Fresh fecal pellets were collected at the same time of the day (mid-morning) by placing mice into individual containers, and observing them until ~100–200 mg of feces were deposited. This usually occurred within a few minutes. Each of the 190 samples was weighed immediately after defecation, placed in a sterile DNA-free 2 ml screw cap tube, flash-frozen in liquid nitrogen, and stored at -80°C.

### DNA extraction, amplification, and sequencing

DNA was extracted from the fecal pellets using the QIAamp DNA Stool Mini kit (Qiagen, Valencia, CA). Samples were processed in batches of approximately sixteen, with one extraction control for every eight samples, to monitor environmental contamination. The V3 region of the bacterial 16S rRNA gene was amplified using bar-coded derivatives of primers 338F (ACT CCT ACG GGA GGC AGC AG) and 533R (TTA CCG CGG CTG CTG GCA C), as described in Dethlefsen, 2008 [52]. PCR products were run on 3% agarose gels, bands excised, and DNA purified using the QIAquick Gel Extraction kit (Qiagen) according to protocol. PCR products were then further purified as recommended by Roche 454 FLX protocols, using AMPure magnetic beads (Agencourt, Beckman Coulter, Danvers, MA)**.** DNA was quantified using the Picogreen Quant-iT dsDNA Assay Kit, High Sensitivity (Invitrogen, Carlsbad, CA) on a Typhoon scanner (GE Healthcare Life Sciences, Piscataway, NJ) in 96 well plates, and then pooled at equimolar concentrations. Samples were submitted to the Duke University ISC sequencing center for pyrosequencing on the Genome Sequencer FLX system (Roche, CA) according to 454 FLX protocols.

### Technical Replicates

Biological and technical replicates of the same microbial community were analyzed to determine the precision of our measurements. The replicates consisted of biological replicates—fecal pellets that were split prior to DNA extraction and then extracted separately—to assess variation within a fecal sample (n = 2), extraction replicates—samples that were split after homogenization but before DNA extraction to assess variation due to the extraction protocol (n = 4), run replicates—samples given the same barcode and run on different sequencing runs to measure variation between sequencing runs (n = 5), barcode replicates—samples given two different barcodes and run on the same sequencing run to assess barcode to barcode variation (n = 4), and barcode/run replicates—samples given different barcodes and run on different sequencing runs (n = 5).

### OTU and taxonomic assignment

Pyrosequencing reads were subjected to quality control filters, which specified that there must be two correct sample keys present, 0 or 1 ambiguous nucleotides present, and a target region > 130 nucleotides in length. Sample keys and primer sequences were trimmed from the read, as described in Dethlefsen et al. [52]. 2,046,788 reads passed the quality control parameters and were further analyzed through the Quantitative Insights Into Microbial Ecology (QIIME) pipeline (http://qiime.sourceforge.net/) [53]. Briefly, sequences were binned into Operational Taxonomic Units (OTUs) using a similarity threshold of 97% and a customized reference database derived from the Greengenes 12–10 release clustered at 99% sequence identity threshold (available upon request), allowing for new clusters. Chimeras were identified and removed using UCHIME [54]. OTU-representative sequences were aligned and masked using the Lane mask, and a phylogenetic tree was built using the FastTree software implemented in QIIME. Taxonomy was assigned to each OTU-representative sequence in QIIME using the Ribosomal Database Project (RDP) classifier, a curated Greengenes reference database, and a confidence score of at least 80%. All OTUs with only one read or only seen in one sample were removed. The final dataset contained 1,849,990 reads, 190 samples (average number of reads per sample was 9736, SE = 529) (S1 Fig), and 5,784 OTUs. The pyrosequencing reads were deposited at MG-RAST under accession numbers 4526254.3 to 4526535.3 with project ID 4928.

### Data analysis

All analyses were performed on non-rarefied data, except for alpha diversity measures for which samples with more than 5400 reads were rarified to 5400 reads using QIIME. Phylogenetic trees and alpha diversity metrics were calculated using the phyloseq R package [55]. Co-occurrence analysis were conducted using R. Community comparisons were performed using weighted UniFrac distances [56] and principal coordinates analysis in phyloseq. Biplots were generated in R/phyloseq using a simplified dataset consisting of the 100 most abundant OTUs, using a Bray-Curtis distance method and an NMDS ordination. R scripts are available as an R markdown file (S1 File) and html output (S2 File). Since many of the taxa were present in all mice but differed in abundance in different mice, we used weighted UniFrac distances to capture this aspect of variation. To determine the statistical significance of differences in average pairwise weighted UniFrac distances, Student’s t-tests were used. Slopes of UniFrac distance over time and number of OTUs over time were analyzed in GraphPad Prism. PERMANOVA (PrimerE) was used for non-parametric analysis of variation on individual and nested factors to determine statistical significance and to estimate components of variation using a mixed model. We used a mixed model that treated all factors as random, and nested time (different samples from same mouse) within individual, individual within cage, and cage within shipment. This model used permutation of residuals under a reduced model, partial sum of squares, and 999 permutations. The individual factor tests used the same parameters except unrestricted permutation of raw data. Heatmaps to display the relative abundance of the most abundant OTUs were constructed using Java Treeview (http://jtreeview.sourceforge.net/).

## Results

### Variation in phylogenetic composition of fecal microbiota in a genetically homogeneous population of mice

**Fig 1:**
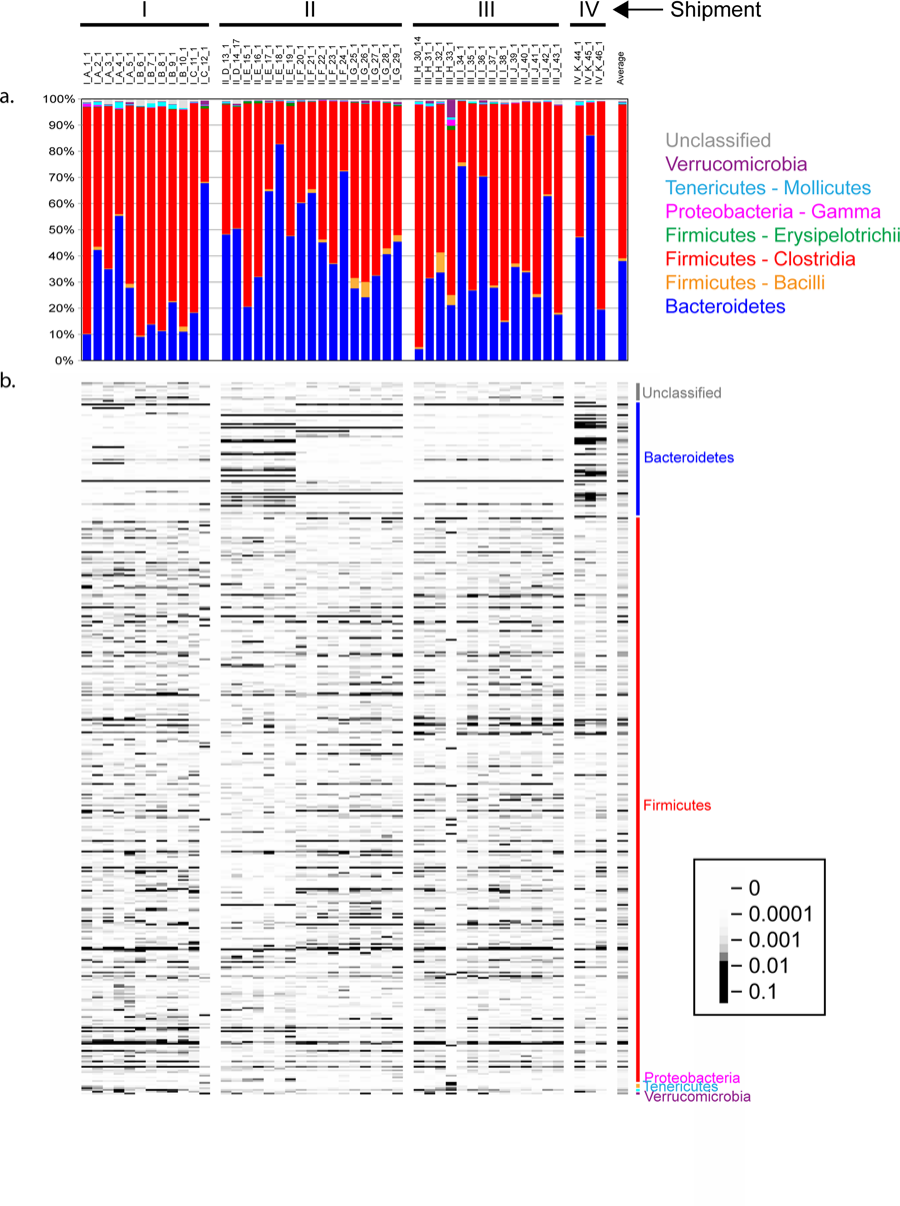
Variation in relative abundances of bacterial taxa from fecal microbial communities of 46 adult healthy mice, ordered per shipment group. A single time point from each mouse (the first collected) is presented. The column on the right shows the average of all 46 samples. (a) Relative abundances of phyla and classes. (b) Heatmap showing the relative abundances of the 400 most abundant OTUs. Phylum assignments of these OTUs are shown along the right side of the heatmap. Key on the right indicates the correspondence of the gray values to the relative OTU abundance.

Overall, the fecal microbiota of the 46 healthy 129X1/SvJ female mice in this study was primarily composed of taxa from the *Bacteroidetes* and *Firmicutes* phyla, with additional contributions from *Tenericutes*, *Verrucomicrobia*, *Proteobacteria*, *Cyanobacteria*, *Actinobacteria*, *Fusobacteria*, *Synergistetes*, and TM7 (Fig 1, S2 Fig). Within these phyla, the greatest diversity was found in *Firmicutes* (Fig 1B, S2 Fig). These results are similar to those of other studies of laboratory mice [10,23,38].

Despite inclusion of only female mice from a single inbred strain, which were fed the same diet and housed under the same environmental conditions, we found a high level of variation in the phylogenetic composition of the fecal microbiota among different mice (Fig 1). At the phylum level, we found members of *Firmicutes*, *Bacteroidetes*, and *Tenericutes* in all mice, whereas members of *Verrucomicrobia*, *Proteobacteria*, *Cyanobacteria*, *Actinobacteria*, and *Fusobacteria* were detected in 83%, 79%, 17%, 6%, and 4% of mice, respectively. The relative abundance of phyla also varied among mice. For example, the proportion of *Bacteroidetes* varied from 4% to 86% across this mouse population (Fig 1A). The majority of OTUs were assigned to the Phylum *Firmicutes* and the presence of individual OTUs varied across individuals (Fig 1B). There were only two OTUs (one in the Family *Lachnospiraceae*, and one in the Genus *Anaeroplasma* **)** that were present in all mice, and only 4% of all OTUs were found in at least half of the mice. While there were no clear patterns of variation in the *Firmicutes*, among the *Bacteroidetes* there appeared to be groups of OTUs that differed in abundance between cages and shipment groups (Fig 1B). We found evidence of taxon co-occurrence and exclusion, which can suggest cooperative or competitive interactions (S3 Fig). Because taxon pairs that co-occur may share similar ecological characteristics, co-occurrence patterns can be valuable in determining traits of taxa that co-occur with well-characterized organisms [57].

### Temporal variation in phylogenetic composition of fecal microbiota

**Fig 2:**
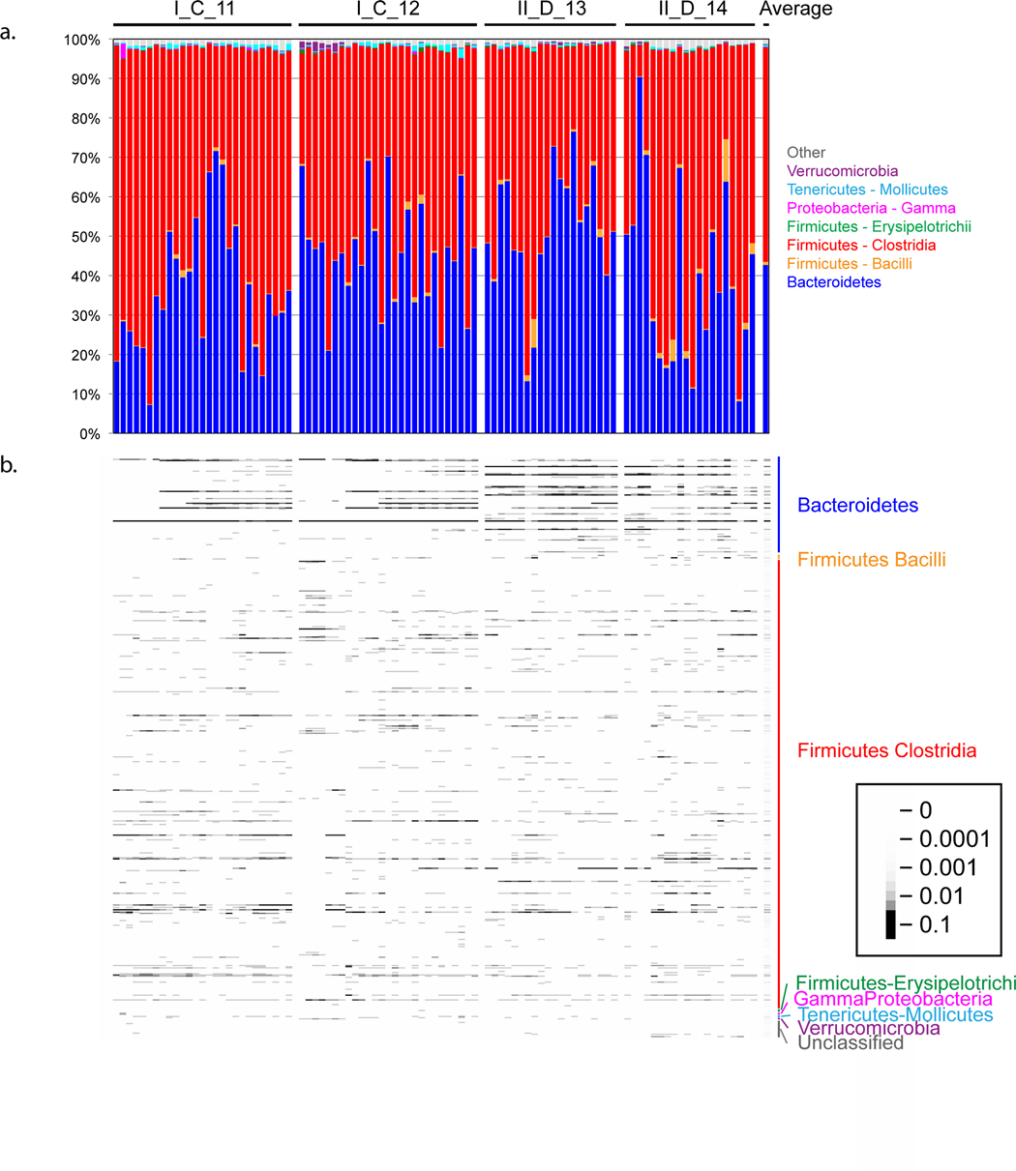
Variation in fecal bacterial diversity over time. Relative taxa abundances are shown for four mice sampled over 200 days. Timepoints are shown in chronological order for each mouse. The column on the right shows the average of all 46 samples. (a) Relative abundances of phyla and classes. (b) Heatmap showing the relative abundances of the 400 most abundant OTUs in the four mice sampled for more than 200 days. Phylum and class assignments of these OTUs are shown along the right side of the heatmap. Key on the right indicates the correspondence of the gray values to the relative OTU abundance.

To determine the variation in individual mice over time we sampled 4 mice for over 200 days. We found large differences in the relative abundance of the major phyla within an individual mouse; for example, in mouse II_D_14 the relative abundance of *Bacteroidetes* ranged from less than 10% to greater than 90% (Fig 2A). We also found shifts in abundance at the OTU level within a mouse over time, including an increase in some *Bacteroidetes* OTUs (Fig 2B). To determine if the overall number of OTUs increased over time, we calculated the number of OTUs present in all samples from these four time courses (data rarefied to 5400 reads per sample). We found that the number of OTUs increased over the first 50 days (slope = 3.5 +/- 1.2; p-value = 0.005) (S4A Fig) When the data were separated by mouse, we found that this trend was significant in three of the four mice, with the other one having a positive but non-significant slope (S4B Fig). Measures of alpha diversity and evenness showed similar patterns (S4C–S4H Fig).

### Inter-individual variation in microbiota composition is greater than intra-individual variation over time

**Fig 3:**
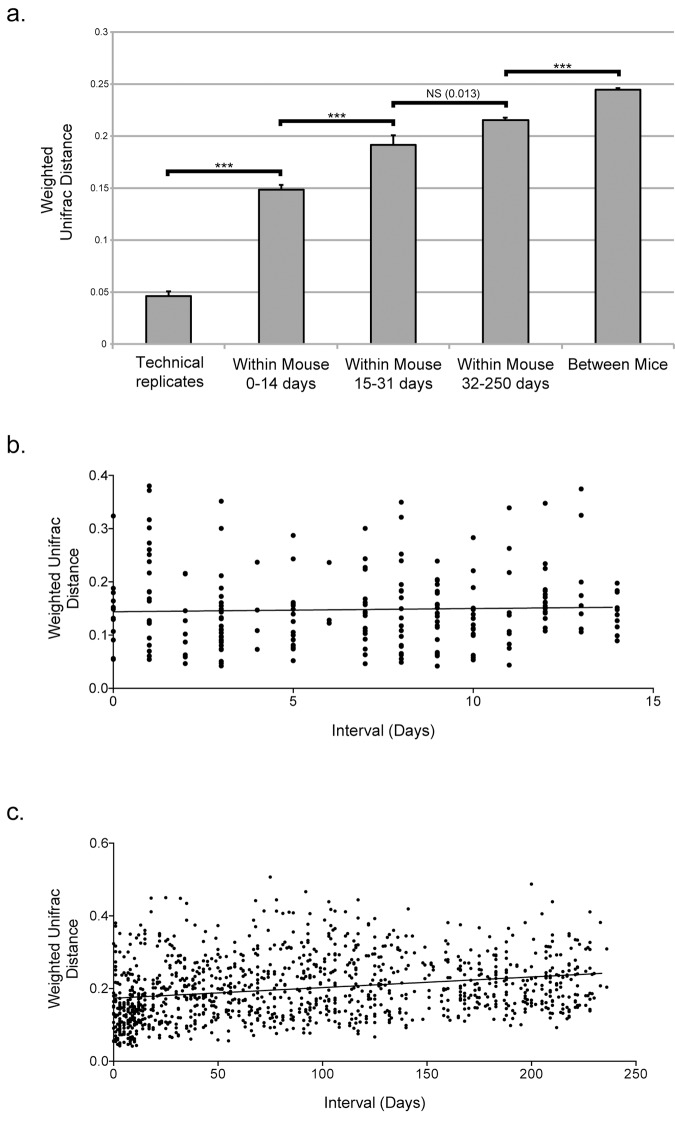
Comparison of variation within and between mice. (a) The average pairwise weighted UniFrac distances between technical replicates (includes all replicates—biological, extraction, sequencing run, and barcode), within 43 individual mice over less than two weeks (14 days), within four individual mice over 15–31 days, within four individual mice over 32–250 days, and among all different healthy mice. Bar height indicates mean; thin lines at the top indicate the standard error. All categories were significantly different from the technical replicates. ***, p<0.001. NS, non-significant. (b) Average pairwise weighted UniFrac distances between samples collected from the same mice at different times, plotted against time interval between sampling, for up to 14 days. The line represents the linear regression slope. (c) Same as (b) except that time intervals are as long as 236 days.

In order to characterize the degree of variation among fecal communities in different mice, we quantified the variation in phylogenetic composition of the microbiota among different mice by calculating the average pairwise weighted UniFrac distance between fecal microbial communities. The average distance between mice was 0.19 (SE = 0.001), which was significantly greater than the average pairwise UniFrac distance among the replicates (0.04, SE = 0.004) (p<0.001) (Fig 3A), indicating that mice have distinct, individualized communities.

We then characterized the variation within an individual mouse over time. Samples collected at multiple time-points were available from 43 of the 46 mice, allowing for an analysis of temporal stability in nearly all mice included in this study. The average pairwise weighted UniFrac distance between samples from an individual mouse over periods of time up to two weeks in duration was 0.12 (SE = 0.004), which was significantly greater than the average weighted UniFrac distance for pairs of replicates as defined above (0.04, SE = 0.004) (p<0.001) (Fig 3A). To determine if there was evidence of temporal autocorrelation, i.e., closely-timed samples having greater similarity to each other than samples produced further apart in time, we examined the average pair-wise weighted UniFrac distance between samples from the same individual mice across the population, as a function of the time interval separating the samples. Among the samples from the 46 mice sampled for as long as 2 weeks, there was no significant change in UniFrac distance relative to time interval (Fig 3B). However, when samples were included from longer time periods, we did find a significant increase in distance as time between sampling increased (p<0.0001) (Fig 3C). This could be due to stochastic drift between the communities [58].

To determine if individual mice have distinct microbial communities over time, we compared the magnitude of the variation in microbiota composition within individual mice over time to the magnitude of variation among mice in the population (Fig 3A). For the four mice from which multiple samples were collected over more than 200 days, the average pair-wise weighted UniFrac distance between different samples from an individual mouse was significantly greater than the average distance between different samples from an individual mouse within the larger population of 46 animals from whom samples were collected for only approximately 2 weeks (p<0.001). However, the average pair-wise weighted UniFrac distance between samples from an individual mouse over any time interval was significantly less than the average pairwise distance between samples from different individuals (p<0.001). Using PERMANOVA (a non-parametric form of ANOVA), we found that the individual was a significant source of variation in the data (p = 0.001). This suggests that despite the variation over time, individual laboratory mice maintain distinct microbial communities.

### Shipment group accounts for some cage-to-cage variation but not inter-individual differences

**Fig 4:**
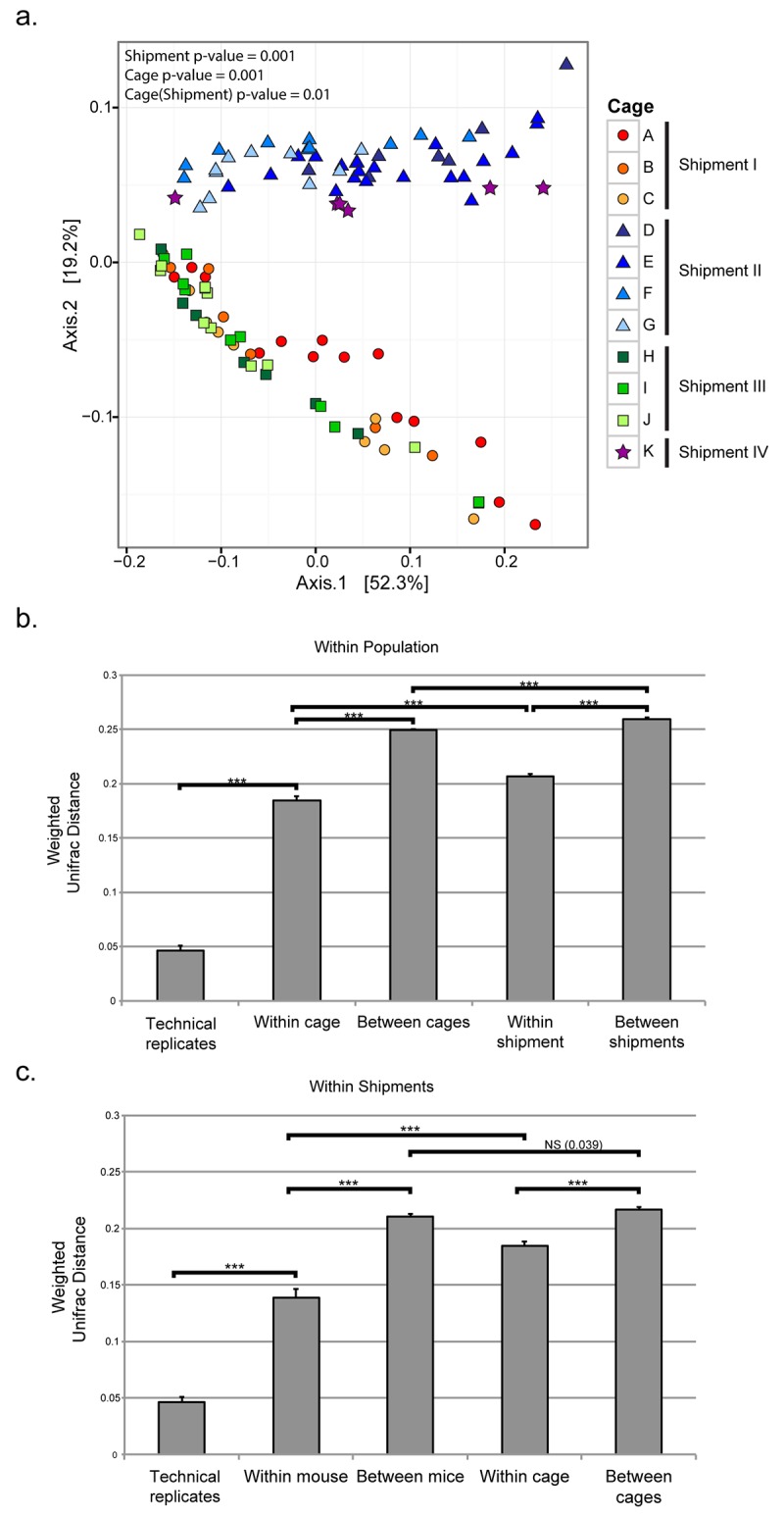
Comparison of variation within and among cages and shipment groups. (a) Principal coordinate plot of weighted UniFrac distances among microbiota from 46 mice sampled at 1–4 time points over a period of two weeks. Shapes of data points correspond to shipment group and colors of data points correspond to cage. Shipment p value was calculated using PERMANOVA on the factor of shipment group. Cage p value was calculated using PERMANOVA on the factor of cage. Cage(Shipment) p value was calculated using a PERMANOVA model where the factor of cage was nested within the factor of shipment group. (b) The average pairwise weighted UniFrac distance of samples from different mice within the same cage, between samples from mice in different cages, between samples from mice within the same shipment group, and between samples from mice in different shipment groups, across the entire population of mice. The average distance for each category was statistically significantly different from that of the technical replicates (includes all replicates—biological, extraction, sequencing run, barcode, and barcode/run). *** indicates p<0.001. ** indicates p<0.01. NS indicates p>0.05. (c) The average pairwise weighted UniFrac distance between samples from the same mouse, between samples from different mice, between samples from mice within the same cage, and between samples from mice in different cages within each shipment group. *** indicates p<0.001. NS indicates p>0.05.

We hypothesized that mice received from the vendor in the same shipment would have more similar microbiota than mice received in different shipments. The mice used in this study were obtained from Jackson Laboratories, where mice of approximately the same age (3 weeks, +/- 3 days) from multiple litters were pool-weaned and maintained in the same weaning group prior to shipment (see Methods). While we do not know which, if any, of the mice were littermates (this information was not available), we do know that mice from the same shipment were housed together during weaning, the crucial period during which it is believed that the microbiota begins to develop a more adult-like profile [59,60]. Fecal specimens from mice of the same shipment tended to cluster on a PCoA plot where the two principal components (PC1 and PC2) together accounted for 70% of the variability in the data (especially shipments I and III, versus II and IV) (Fig 4A). Bray-Curtis distance plots showed similar patterns and biplots suggest the differences in shipments are explained by OTUs (S5 Fig). PERMANOVA indicated that microbiota composition differences among mice from different shipment groups were statistically significant (p = 0.001). Furthermore, the average pair-wise weighted UniFrac distance between specimens from mice of the same shipment was significantly lower than the average distance between specimens from mice of different shipments (Fig 4B). Strikingly, the range of microbiota compositions from shipments I and III were similar to each other, and likewise the ranges of microbiota compositions from shipments II and IV were similar to each other, even though each of these pairs of shipments was separated in time by several months.

Mice from the same cage also share microbiota through coprophagy; therefore, we speculated that the microbiota of mice housed in the same cage would be more similar to each other than would microbiota of mice from different cages. Other investigators have shown evidence for this type of “cage effect” [10,15,20,29,45]. In our experiments, mice were housed in the same cage for at least one to two weeks prior to the initiation of sample collection. Given the lack of significant temporal autocorrelation for samples taken from the same mouse over intervals as short as a few days (Fig 2A), this co-housing period should be sufficient for microbiota composition to stabilize if cage-mates were influencing the microbiota of one another. Using PERMANOVA, cage was a statistically significant factor (p = 0.001); the microbiota of mice from the same cage were significantly more similar to one another than the microbiota of mice from different cages, based on weighted UniFrac distance (Fig 4B).

Since there was such a strong shipment group effect and mice in the same cage were also from the same shipment, we asked whether differences between mice from different shipment groups were responsible for the differences seen among individuals from different cages. To determine if mice from the same cage had more similar microbiota than mice from different cages but from the same shipment group, we used a PERMANOVA model in which individual was nested within cage and cage was nested within shipment group, and found that the cage effect was reduced, although cage remained a statistically significant factor in determining microbiota composition (p = 0.009). Within a shipment group, the average pair-wise weighted UniFrac distance between microbiota of mice in different cages remained significantly greater than that between microbiota of mice within a cage, but this difference was reduced compared to that in the entire mouse population (Fig 4B and 4C). This suggests that separation of pool-weaned mice into different cages has an effect on the composition of the microbiota.

The shipment group effect also could have inflated individual-to-individual differences. Therefore, we examined whether intra-mouse variation over time was less than the variation among mice over time for mice from the same shipment. Using a PERMANOVA model where individual was nested within cage, and cage within shipment, we found that the effect of individual was still statistically significant (p = 0.001). We also calculated the pairwise weighted UniFrac distances between specimens from mice from the same shipment. We found that the average weighted UniFrac distance between mice within the same shipment was 0.17, which was significantly greater than the variation within a mouse (p<0.001) (Fig 4C). This indicates that there are distinct differences among the microbiota of individual mice, even within a single shipment group.

### Relative contributions of environmental factors to patterns of fecal microbial diversity

To determine the relative contributions of different environmental factors, such as cage and shipment, we performed PERMANOVA with a model containing multiple nested factors. Using all samples from all mice and weighted UniFrac distances, we found that 23% of the variation in intestinal microbiota composition was explained by changes within the fecal microbiota of a mouse over time, 18% by shipment group, 14% by cage, and 12% by persistent differences among individual mice. The rest of the variation (33%) was residual in the model. In this model, time (sample), individual, cage, and shipment group were all random effects, and time (sample) was nested within individual, individual nested within cage, and cage nested within shipment group. These results suggest that the environment in which a mouse is weaned contributes strongly to the phylogenetic composition of the adult microbiota.

## Discussion

Using inbred female mice of approximately the same age from the same vendor that were fed the same diet and housed in the same environment, we examined variation in the taxonomic composition of the fecal microbiota among different mice and within individuals over time. Our data indicate significant variation in the microbiota among individual mice under these controlled conditions. This suggests that factors other than genetics, diet, and gross environmental features have a significant impact on the composition of the microbiota. It has been reported that humans or animals who cohabit have microbiota that are more similar than those of non-cohabitating individuals [5,8,10,15]. Animals in contact with each other at the time of weaning also appear to share microbiota [20]. Yet, in our study, when we controlled for founder effects from the environment at the time of weaning and for exposure to the microbiota of other individuals through cohabitation, there was significant variation in community composition among individuals. These findings suggest that there are as yet undetermined factors or stochastic effects that significantly influence and contribute to the distinctness of the composition of an individual’s microbiota. The identity of these factors and the sources of relevant environmental microbiota (source-tracking) are areas where further research is necessary. In this study, we were not able to control for maternal effects since we did not have information about which mice, if any, were littermates. While this is known to be an important factor in the development of the microbiota, we would not expect maternal effects, based on previous work, to account for all of the unexplained variation in our data [20,21,23].

We also found measurable variation in the microbiota of an individual mouse over time. However, this variation was significantly less than that among different mice. In humans, temporal variation is mainly believed to be due to changes in diet or other aspects of the environment. Our findings of significant temporal variation in a controlled environment and constant diet may suggest that diet and environment may play a smaller role in human temporal variation than previously thought and that other factors may be playing a larger role. Alpha-diversity of the fecal microbiota of these mice increased during the first 50 days of laboratory study suggesting that there may be an early period of environmental accommodation and/or ongoing intestinal microbiota assembly following relocation in the early post-weaning period. Some of this variability may have been due to post weaning instability. However, Schloss et al. showed that by 11–17 days post weaning the most dramatic temporal variability of the murine distal gut bacterial communities has ended and these communities resemble a more mature and stable state, suggesting that the effects we found in mice 5–8 weeks old were caused by factors other than weaning [38]. We also found that despite the shared environment and the absence of significant introduction of new OTUs, the microbial communities of mice became more different over time, which could be the results of host-specific selection on the individual communities or stochastic drift. Since these long-term observations of temporal dynamics were performed on only four mice, similar experiments with more mice would be valuable and might provide additional insights.

In our study, shipment group was also highly associated with microbiota composition. The shipment group effect is likely due to the fact that mice from the same shipment had been previously pool-weaned together and therefore shared a similar environment and external source of microbiota at that time. Within the same shipment group, the cage effect was reduced but was still significant. This suggests that for mice that were weaned together later separation and cohabitation can still affect microbiota composition. It also suggests that for efforts to create a population of mice with similar microbiota, mouse co-weaning may have a larger impact, but co-housing may also reduce variation. However, since in this study all mice within a cage derived from the same pool-weaned group, we can not fully discern the effects of co-housing on mice from different pool-weaning groups. Longer periods of co-housing may in fact increase the cage effect [61]. In order to determine more definitively whether weaning group or subsequent cohabitation has a greater effect on the composition of the microbiota in mice, mice from different weaning groups would need to be housed together as adults and for longer periods of time.

These findings suggest caution before assuming that the microbiotas of inbred mice do not vary among individuals. Since many roles of the gut microbiota have only recently been recognized and others may still remain unrecognized, it is possible that differences in the microbiota of inbred mice may be responsible for variation and noise in many kinds of host phenotypes. For example, since the composition of the microbiota and the presence of specific taxa are important for responses to drugs and susceptibility to specific pathogens [3,4,62], inter-individual differences in microbiota could result in differential responses to drugs or to pathogens. Awareness and understanding of these confounding effects will allow investigators to design their experiments so as to control for them.

## Supporting Information

S1 Fig Number of reads per sample. Of note, this histogram includes a discontinuous x-axis.
(TIF)

S2 Fig Phylogenetic tree of OTUs found in 46 mice. The tree displays the 285 OTUs represented by 100 reads or more, and present in at least 5 samples. Abundance of all OTUs by shipment group is shown to the right of the tree. Color of dot represents shipment group (red = I, green = II, blue = III, purple = IV). Abundance is represented by size of dot, shown on a log10 scale. Phyla and class are indicated on the left. Color of taxon label indicates phylum.
(TIF)

S3 Fig Co-occurrence of taxa in mice. Co-occurrence was calculated at the level of genus to determine the probability that a more extreme value of co-occurrence could have been obtained by chance, using the R package “co-occur”. Of 3240 species pair combinations, 892 pairs (27.5%) were removed from the analysis because expected co-occurrence was < 1; 2348 pairs were analyzed. Blue cells indicate genus pairs that were found together in the same sample more often than expected (p<0.05); red cells indicate genus pairs that were found together in the same sample less often than expected (p<0.05). Asterisks indicate p values < 0.00002, after Bonferroni correction for 2348 hypotheses.
(TIF)

S4 Fig Alpha diversity over time. Alpha diversity is displayed across all samples from four time courses at 5400 reads per sample for (a) Observed OTUs, (c) Shannon Diversity Index, (e) Simpson’s Diversity Index, and (g) Shannon Evenness, and by individual mouse for (b) Observed OTUs, (d) Shannon Diversity Index, (f) Simpson’s Diversity Index, (h) Shannon Evenness.
(TIF)

S5 Fig Bi-plot of samples and taxa. (a) NMDS ordination of Bray-Curtis distance among microbiota from 46 mice sampled at 1–4 time points over a period of two weeks. Shapes of data points correspond to shipment group and colors of data points correspond to cage. (b) Bi-plot of samples and 100 most abundant OTUs colored according to genus. Samples are light grey and shape correspond to shipment.
(TIF)

S1 File R Markdown file for R script corresponding to bi-plot analysis. (RMD)

S2 File HTML output for R script corresponding to bi-plot analysis. (HTML)
