# Supplementary figures and images for: Variation in Taxonomic Composition of the Fecal Microbiota in an Inbred Mouse Strain across Individuals and Time
Source: PLoS One. 2015 Nov 13;10(11):e0142825. doi: 10.1371/journal.pone.0142825 (PMC4643986; doi:10.1371/journal.pone.0142825)

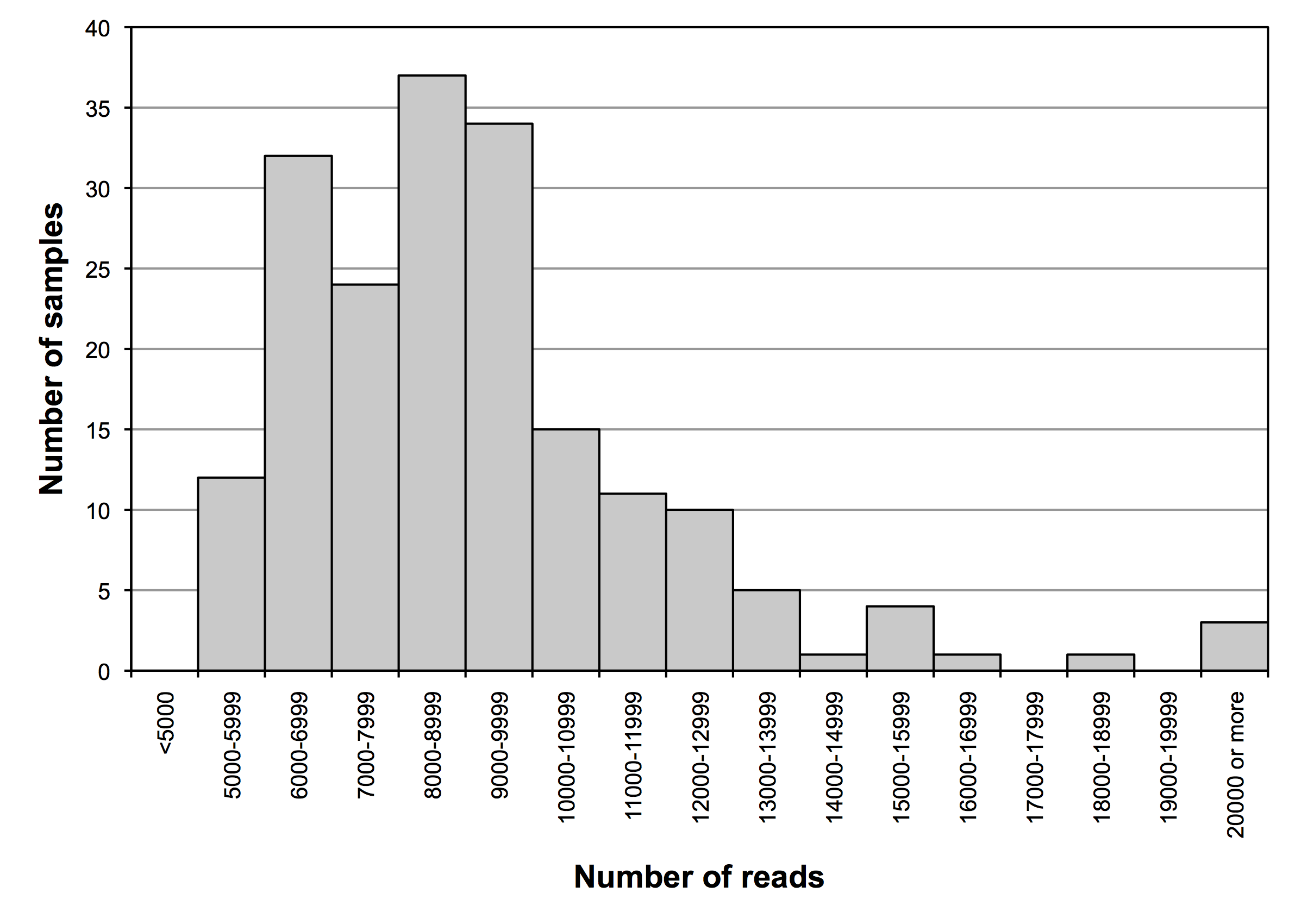

Supplement: S1 Fig — Of note, this histogram includes a discontinuous x-axis. (TIF) [file pone.0142825.s001.tif]

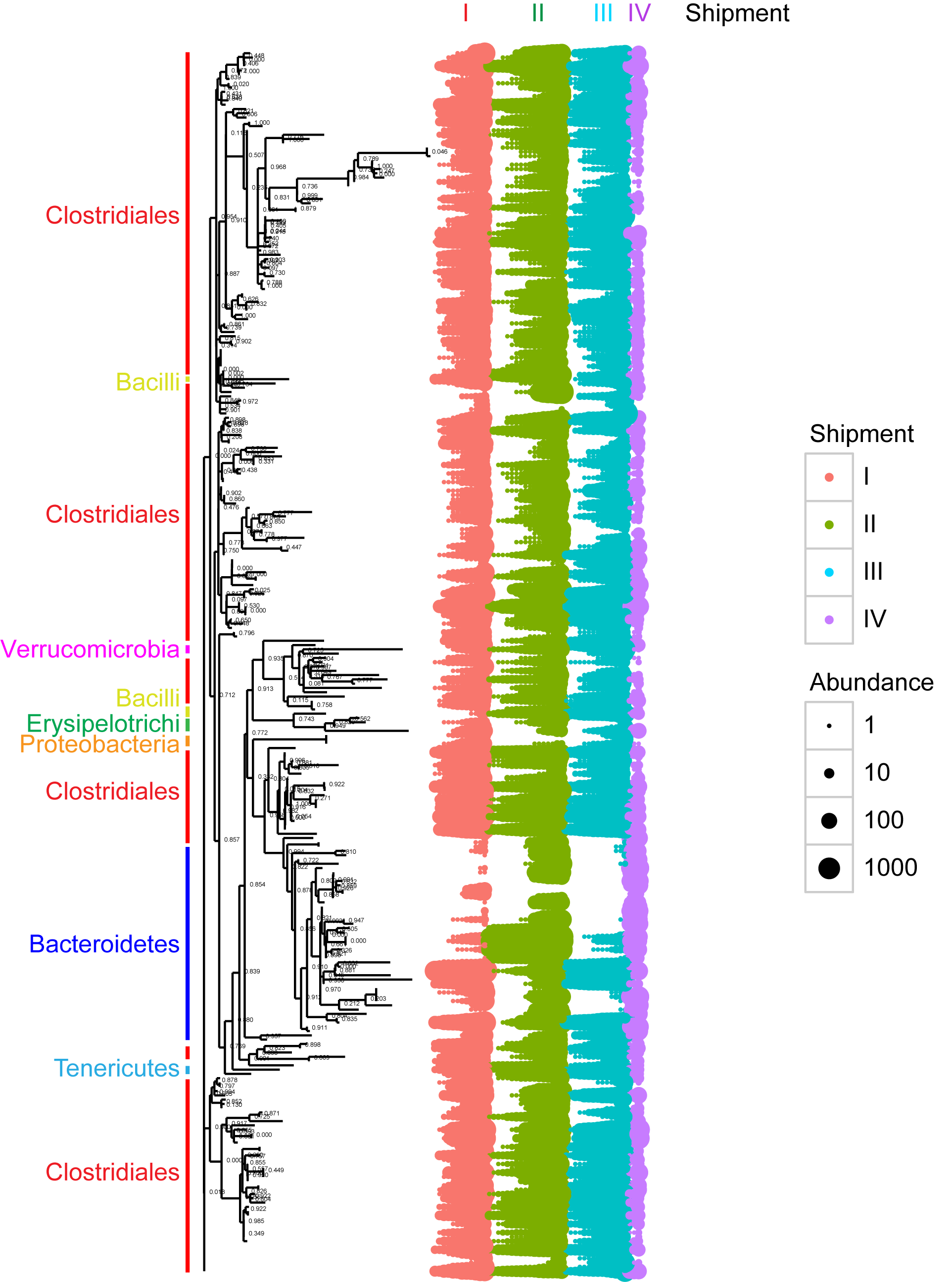

Supplement: S2 Fig — The tree displays the 285 OTUs represented by 100 reads or more, and present in at least 5 samples. Abundance of all OTUs by shipment group is shown to the right of the tree. Color of dot represents shipment group (red = I, green = II, blue = III, purple = IV). Abundance is represented by size of dot, shown on a log10 scale. Phyla and class are indicated on the left. Color of taxon label indicates phylum. (TIF) [file pone.0142825.s002.tif]

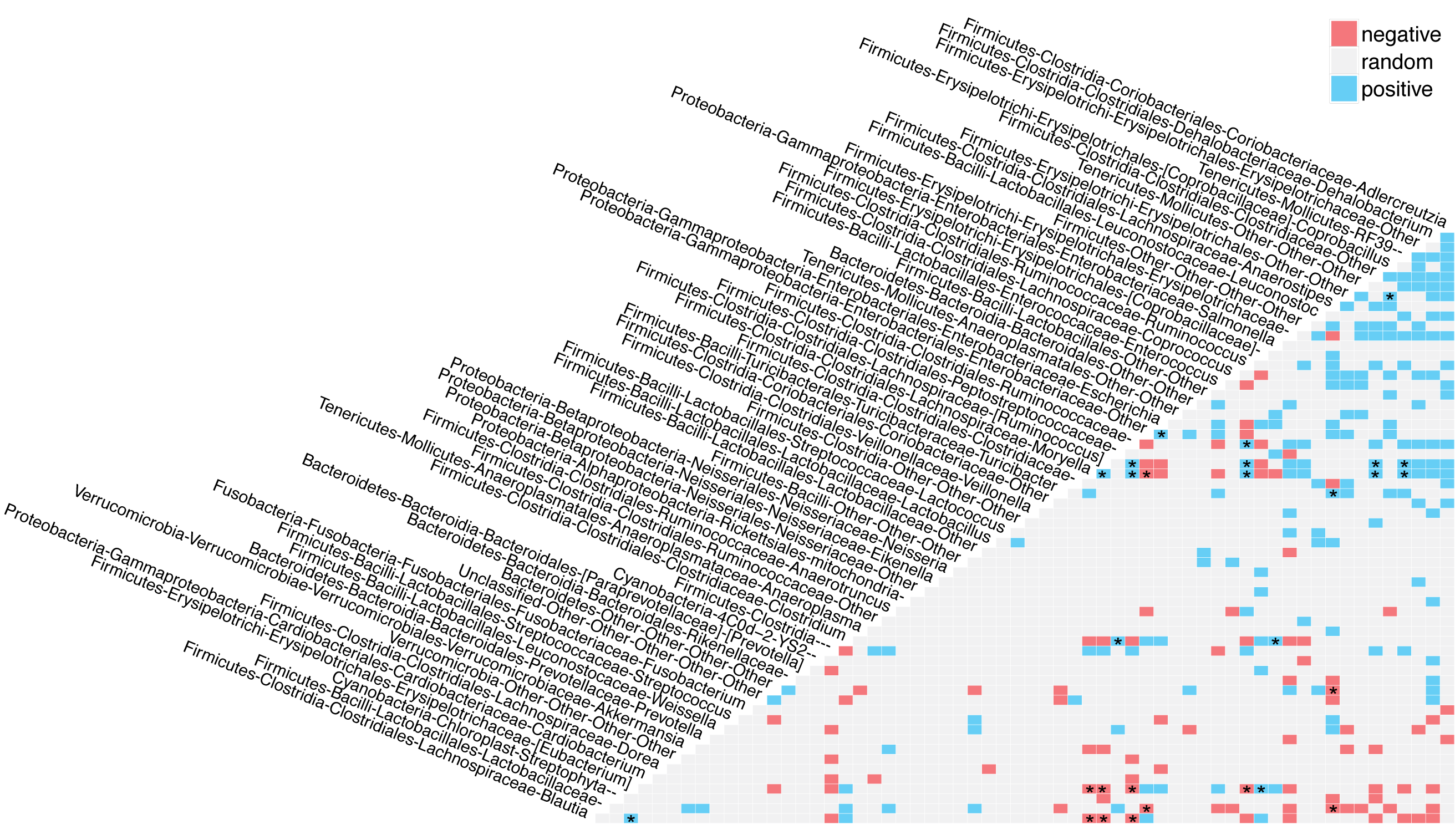

Supplement: S3 Fig — Co-occurrence was calculated at the level of genus to determine the probability that a more extreme value of co-occurrence could have been obtained by chance, using the R package “co-occur”. Of 3240 species pair combinations, 892 pairs (27.5%) were removed from the analysis because expected co-occurrence was < 1; 2348 pairs were analyzed. Blue cells indicate genus pairs that were found together in the same sample more often than expected (p<0.05); red cells indicate genus pairs that were found together in the same sample less often than expected (p<0.05). Asterisks indicate p values < 0.00002, after Bonferroni correction for 2348 hypotheses. (TIF) [file pone.0142825.s003.tif]

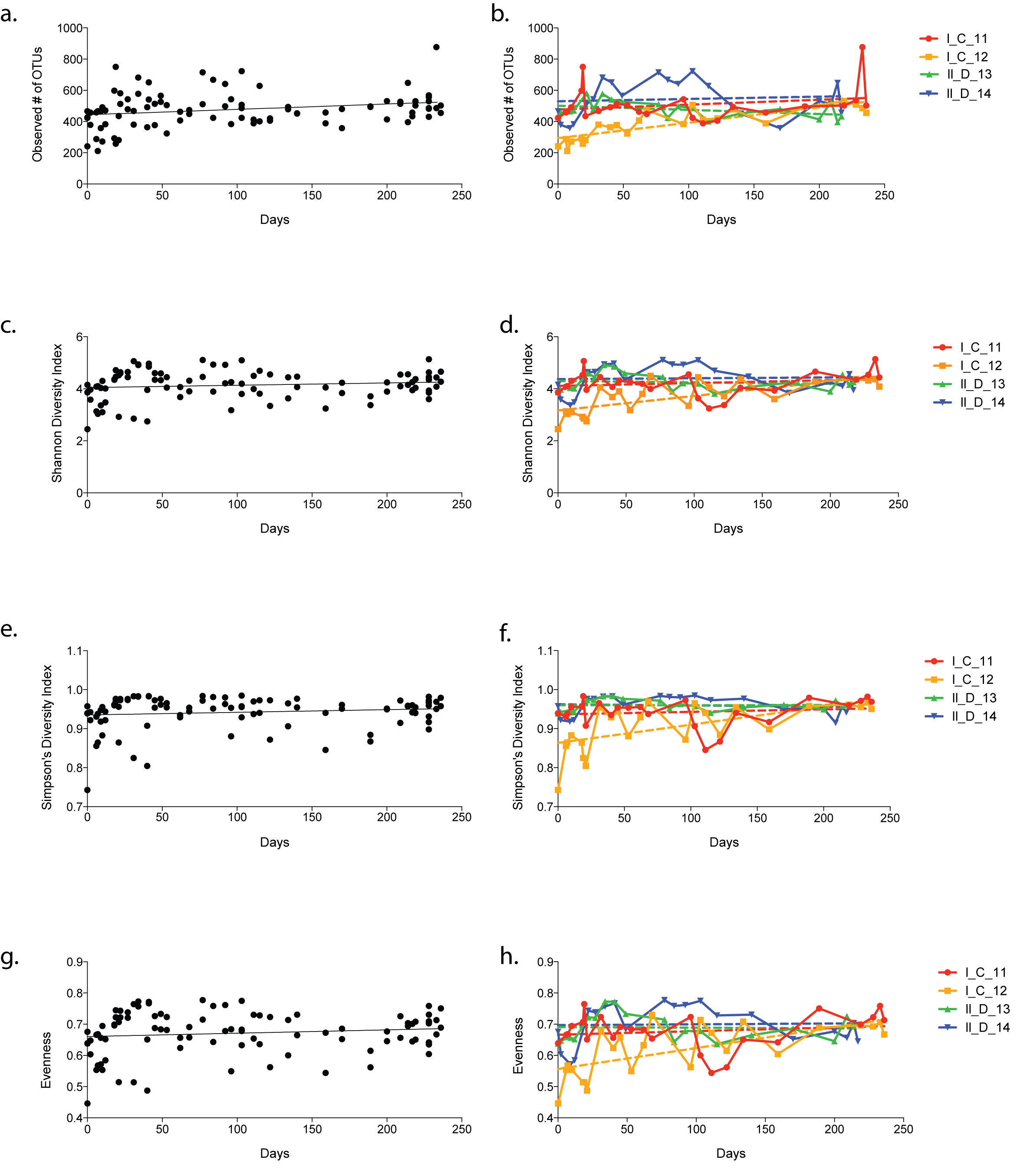

Supplement: S4 Fig — Alpha diversity is displayed across all samples from four time courses at 5400 reads per sample for (a) Observed OTUs, (c) Shannon Diversity Index, (e) Simpson’s Diversity Index, and (g) Shannon Evenness, and by individual mouse for (b) Observed OTUs, (d) Shannon Diversity Index, (f) Simpson’s Diversity Index, (h) Shannon Evenness. (TIF) [file pone.0142825.s004.tif]

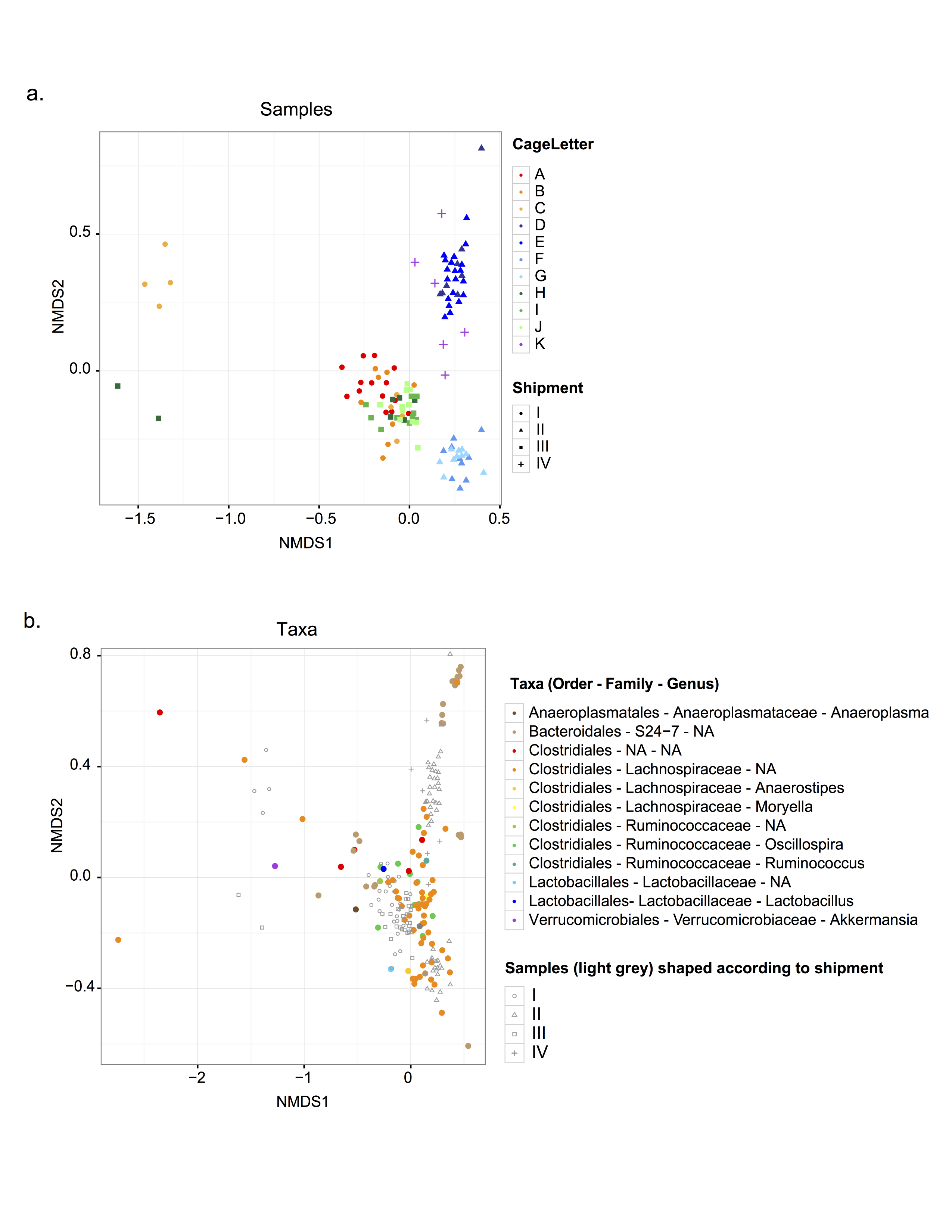

Supplement: S5 Fig — (a) NMDS ordination of Bray-Curtis distance among microbiota from 46 mice sampled at 1–4 time points over a period of two weeks. Shapes of data points correspond to shipment group and colors of data points correspond to cage. (b) Bi-plot of samples and 100 most abundant OTUs colored according to genus. Samples are light grey and shape correspond to shipment. (TIF) [file pone.0142825.s005.tif]
