# Supplementary material for: Variation in Taxonomic Composition of the Fecal Microbiota in an Inbred Mouse Strain across Individuals and Time
Source: PLoS One. 2015 Nov 13;10(11):e0142825. doi: 10.1371/journal.pone.0142825 (PMC4643986; doi:10.1371/journal.pone.0142825)
Supplement: S2 File — (HTML) [file pone.0142825.s007.html]

# Yana Hoy mouse set - June 2015 - Elisabeth Bik

# Phyloseq analysis of 16S rRNA amplicon (V3 region) 454-FLX generated sequence read dataset on 46 mice. OTU tables, taxonomy assignments, and phylogenetic tree were generated in QIIME.

# # Most mice were sampled 1-4 times in 2 weeks; 4 mice were followed for over 200 days (20-27 timepoints per mouse). Total data set has 190 samples and 13 additional replicates. We only retained OTUs that had more than 1 read (so removed singletons), and seen in more than 1 sample. This reduced number of OTUs from 11,396 to 5784.

# Libraries and initialization:

```
R.Version()
```

```
## $platform
## [1] "x86_64-apple-darwin10.8.0"
## 
## $arch
## [1] "x86_64"
## 
## $os
## [1] "darwin10.8.0"
## 
## $system
## [1] "x86_64, darwin10.8.0"
## 
## $status
## [1] ""
## 
## $major
## [1] "3"
## 
## $minor
## [1] "1.1"
## 
## $year
## [1] "2014"
## 
## $month
## [1] "07"
## 
## $day
## [1] "10"
## 
## $`svn rev`
## [1] "66115"
## 
## $language
## [1] "R"
## 
## $version.string
## [1] "R version 3.1.1 (2014-07-10)"
## 
## $nickname
## [1] "Sock it to Me"
```

```
library("phyloseq")
```

```
## Warning: no function found corresponding to methods exports from
## 'S4Vectors' for: 'width'
```

```
packageVersion("phyloseq")
```

```
## [1] '1.9.13'
```

```
library("ggplot2")
packageVersion("ggplot2")
```

```
## [1] '1.0.0'
```

```
library("ape")
packageVersion("ape")
```

```
## [1] '3.1.4'
```

```
library("DESeq2")
```

```
## Loading required package: GenomicRanges
## Loading required package: BiocGenerics
## Loading required package: parallel
## 
## Attaching package: 'BiocGenerics'
## 
## The following objects are masked from 'package:parallel':
## 
##     clusterApply, clusterApplyLB, clusterCall, clusterEvalQ,
##     clusterExport, clusterMap, parApply, parCapply, parLapply,
##     parLapplyLB, parRapply, parSapply, parSapplyLB
## 
## The following object is masked from 'package:stats':
## 
##     xtabs
## 
## The following objects are masked from 'package:base':
## 
##     anyDuplicated, append, as.data.frame, as.vector, cbind,
##     colnames, do.call, duplicated, eval, evalq, Filter, Find, get,
##     intersect, is.unsorted, lapply, Map, mapply, match, mget,
##     order, paste, pmax, pmax.int, pmin, pmin.int, Position, rank,
##     rbind, Reduce, rep.int, rownames, sapply, setdiff, sort,
##     table, tapply, union, unique, unlist
## 
## Loading required package: IRanges
## 
## Attaching package: 'IRanges'
## 
## The following object is masked from 'package:phyloseq':
## 
##     distance
## 
## Loading required package: GenomeInfoDb
## Loading required package: Rcpp
## Loading required package: RcppArmadillo
```

```
packageVersion("DESeq2")
```

```
## [1] '1.4.5'
```

```
library(plotrix)
```

```
## Warning: package 'plotrix' was built under R version 3.1.3
```

```
packageVersion("plotrix")
```

```
## [1] '3.5.12'
```

```
# source("http://bioconductor.org/biocLite.R")
# biocLite("vsn")
library("plyr")
```

```
## 
## Attaching package: 'plyr'
## 
## The following objects are masked from 'package:IRanges':
## 
##     desc, rename
```

```
packageVersion("plyr")
```

```
## [1] '1.8.1'
```

```
library("doParallel")
```

```
## Loading required package: foreach
## Loading required package: iterators
```

```
packageVersion("doParallel")
```

```
## [1] '1.0.8'
```

```
library("foreach")
packageVersion("foreach")
```

```
## [1] '1.4.2'
```

```
theme_set(theme_bw())
```

# Choose your workspace (change to your own environment)

setwd(“/Users/elies/Desktop/”) setwd(“/Users/EliesBik/Desktop/”)

# Creating a new phyloseq object (can skip and import once done; in that case, jump to the START HERE part)

# Import OTU table (OTU table contains data from 190 samples, plus 13 replicates; 46 unique mice; 4 of these were followed for several months; for other animals 1 to 4 timepoints)

OTUtable <- import\_biom(“Yana\_OTUtable\_SelectionPlusReps\_SingleReadsSingleSamplesRemoved\_13Jun2015.biom”) colnames(tax\_table(OTUtable)) <- c(“Kingdom”, “Phylum”, “Class”, “Order”, “Family”, “Genus”, “Species”)

# import mapping file used in Qiime

mapfile <- import\_qiime(mapfilename=“Yana\_Selection190samples\_13reps\_Mapping\_June2015.txt”)

# importing the tree generated in Qiime

treefile <- import\_qiime(treefilename=“Yana\_Pruned\_OTUs\_5784.tre”)

# create a single phyloseq object with a short name

PS <- merge\_phyloseq(OTUtable, mapfile, treefile) print(PS)

# saving the phyloseq object, so next time we can quickly import it

save(PS, file = “Yana\_prunedUninfectedSamples\_June2015.RData”)

# START HERE: Import the data file (if already created previously)

load(“Yana\_prunedUninfectedSamples\_June2015.RData”) print(PS)

# Look at distribution of the number of samples in which each taxa is observed:

otab <- as(otu\_table(PS), “matrix”) # Taxa are rows present\_absent <- (otab > 0) nsamples <- apply(present\_absent, 1, sum) hist(log(nsamples), 60)

# Make selection of maximum of first 4 timepoints (excluding later timepoints for 4 animals), call this ps4

ps4 <- subset\_samples(PS, FourTPs==“1”) print(ps4)

# Make selection of only first timepoints of each animal (n=46) (st, single timepoints)

st <- subset\_samples(ps4, Days==“0”) print(st)

# Plot a tree of the OTUs found in first timepoints for each animal - need to reduce the dataset; otherwise too many OTUs to make a nice graph

# First, reduce number of OTUs by only selecting the most abundant ones. str, single timepoints reduced number of OTUs

str <- prune\_taxa(nsamples > 5, st) # Found in at least 5 samples print(str) str <- prune\_taxa(taxa\_sums(str) > 100, str) # More than 100 reads across all samples print(str) head(taxa\_sums(str), 20)

# This reduced number of OTUs from 5784 to 285, which is much better for tree plotting.

# One could further reduce number of otus by tip glom - in this case, reduced from 1362 to 313 taxa - better for tree visualization (146 if h=0.2) - for tree building

# pstg <- tip\_glom(ps, h=0.2)

# print(pstg)

# hist(log(taxa\_sums(pstg)), 60)

# Plot a tree on simplified data - 285 most abundant OTUs - without applying the tip glom

plot\_tree(str, color = “ShipmentLatin”, justify = “left”, base.spacing = 0.02, label.tips=“Phylum”, title=“285 OTUs shipment Presence”) plot\_tree(str, color = “ShipmentLatin”, justify = “left”, size = “Abundance”, sizebase=10, label.tips=“Genus”, base.spacing = 0.02, plot.margin=0.5, title=“285 OTUs shipment 10log”, text.size=1) plot\_tree(str, label.tips=“Family”) plot\_tree(str, color=“Class”) plot\_tree(str, color=“Order”)

# Alpha diversity of first 4 timepoints, split per shipment

print(ps4) alphas <- plot\_richness(ps4, x=“ShipmentLatin”, color=“ShipmentLatin”, measures=c(“Observed”, “Chao1”, “Shannon”), title=“First4TPs colored per shipment”) + geom\_point(size = 4, alpha = 0.8) alphas alphac <- plot\_richness(ps4, x=“CageLetter”, color=“CageLetter”, measures=c(“Observed”, “Chao1”, “Shannon”), title=“First4TPs colored per cage”) + geom\_point(size = 4, alpha = 0.8) alphac

# Beta diversity

# ordination on selection set n=112 samples max 4 timepoints (so excluding the later timepoints from the 4 mice that were followed longer)

# we decided to use the PCoA / weighted unifrac graph for the paper

print(ps4) ord\_NMDS\_bray = ordinate(ps4, “NMDS”, “bray”) plot\_ordination(ps4, ord\_NMDS\_bray, shape=“ShipmentLatin”, color=“CageLetter”, title=“NMDS Bray Curtis”)

ord\_NMDS\_wunifrac = ordinate(ps4, “NMDS”, “wunifrac”) pNWUF <- plot\_ordination(ps4, ord\_NMDS\_wunifrac, shape=“ShipmentLatin”, color=“CageLetter”, title=“NMDS Weighted Unifrac”) + aes(size=2) pNWUF

ord\_PCoA\_wunifrac = ordinate(ps4, “PCoA”, “wunifrac”) pPWUF <- plot\_ordination(ps4, ord\_PCoA\_wunifrac, shape=“ShipmentLatin”, color=“CageLetter”, title=“PCoA Weighted Unifrac”) + aes(size=2) pPWUF

# ordination on selection set n=46 samples only first timepoints, so only 1 timepoint per mouse

print(st) ord\_PCoA\_wunifrac\_st = ordinate(st, “PCoA”, “wunifrac”) pPWUFst <- plot\_ordination(st, ord\_PCoA\_wunifrac\_st, shape=“ShipmentLatin”, color=“CageLetter”, title=“PCoA Weighted Unifrac”) + aes(size=2) pPWUFst

# calculate distance matrix to export

dist <- phyloseq::distance(PS, method=“wunifrac”)

# save distance matrix in table form

# convert the upper triangles to NA which are easy to remove in Excel

# disttb[upper.tri(disttb)] = NA

# but this is not handy because trickier to sort later

disttb = as.matrix(dist) disttb[1:5,1:5]

write.table(disttb, “Yana\_wUnifrac\_DistMat.txt”, sep=“”, quote=F, col.names=NA)

# BIPLOT

# Making a biplot - plot both samples as well taxa to see which taxa are driving clustering according to shipment/cage.

# For the biplot, reduce the number of taxa. Only keep the top 100 OTUs

print(ps4) topO = 100 most\_abundant\_taxa = sort(taxa\_sums(ps4), TRUE)[1:topO] print(most\_abundant\_taxa) # strangely, this prints a list of 200 names - not sure why ps4r = prune\_taxa(names(most\_abundant\_taxa), ps4) print(ps4r) # this lists only 100 taxa, so that is as expected

taxa\_r = sort(taxa\_sums(ps4r), TRUE) print(taxa\_r)

# A biplot cannot be done with Unifrac (because UF is not done on OTUs but on unique parts of a tree). It can be done with Bray Curtis, so that is what we will do here.

ord\_NMDS\_bray = ordinate(ps4r, “NMDS”, “bray”)

# Plotting the samples first:

pNBCs = plot\_ordination(ps4r, ord\_NMDS\_bray, type=“samples”, shape=“ShipmentLatin”, color=“CageLetter”, title=“NMDS Bray Curtis samples”) + aes(size=2) pNBCs

# Plotting the taxa in a separate plot - Genus level is good, but handy to also have the same taxa plot colored to higher taxonomic levels, since we have so many OTUs called “g\_*" - this will help in coloring them manually. pNBCc = plot*ordination(ps4r, ord\_NMDS\_bray, type=”taxa“, color=”Class“, title=”NMDS Bray Curtis Class“) + aes(size=2) pNBCc pNBCo = plot\_ordination(ps4r, ord\_NMDS\_bray, type=”taxa“, color=”Order“, title=”NMDS Bray Curtis Order“) + aes(size=2) pNBCo pNBCf = plot\_ordination(ps4r, ord\_NMDS\_bray, type=”taxa“, color=”Family“, title=”NMDS Bray Curtis Family“, label =”Phylum“) + aes(size=2) pNBCf pNBCg = plot\_ordination(ps4r, ord\_NMDS\_bray, type=”taxa“, color=”Genus“, title=”NMDS Bray Curtis Genus“) + aes(size=2) pNBCg

# Plotting as a true biplot - need to force it to use the same symbols as before, otherwise circle shape gets assigned to display taxa and all shapes are shifted

# Value 1 is an open circle (16 is the closed/filled circle)

pNBCb = plot\_ordination(ps4r, ord\_NMDS\_bray, type=“biplot”, color=“Family”, shape=“ShipmentLatin”, title=“NMDS Bray Curtis biplot”) + scale\_shape\_manual(values = c(20, 16, 17, 15, 4)) pNBCb

# Can also plot biplot using Split function, this forces the axes to stay the same.

pNBCs = plot\_ordination(ps4r, ord\_NMDS\_bray, type = “split”, color = “Genus”, shape = “ShipmentLatin”, label = “Phylum”, title = “NMDS Bray Curtis biplot split”) pNBCs

# Run co-occurrence analysis on different levels

# install.packages(“cooccur”)

library(cooccur)

# Order level (L4) Import a tab delimited OTU table, manually converted into 0 and 1 in Excel

orders <- read.table(file=“Yana\_OTUtable\_PresenceAbsence\_L4\_Order\_13Jun2015.txt”, header = TRUE, sep = “”, row.names=1) is.matrix(orders) is.data.frame(orders)

dim(orders) orders[1:5,1:5]

# Doing the Co-Occurrence analysis on ORDER (L4) Level

cooccur.order <- cooccur(mat=orders,type=“spp\_site”, thresh=TRUE, spp\_names=TRUE)

# different ways for seeing the results

summary(cooccur.order) pair.attributes(cooccur.order) pair.profile(cooccur.order) plot(cooccur.order) print(cooccur.order) prob.table(cooccur.order) pair(cooccur.order, “Verrucomicrobia\_Other\_Other”, all = TRUE)

# Doing Cooccurrence analysis on Family (L5) level

family <- read.table(file=“Yana\_OTUtable\_PresenceAbsence\_L5\_Family\_13Jun2015.txt”, header = TRUE, sep = “”, row.names=1)

dim(family) family[1:5,1:5]

cooccur.family <- cooccur(mat=family,type=“spp\_site”, thresh=TRUE, spp\_names=TRUE)

# different ways for seeing the results - Family Level L5

summary(cooccur.family) pair.attributes(cooccur.family) pair.profile(cooccur.family) plot(cooccur.family) print(cooccur.family) prob.table(cooccur.family)

# Doing Cooccurrence analysis on Genus (L6) level

genus <- read.table(file=“Yana\_OTUtable\_PresenceAbsence\_L6\_GenusLevel\_13Jun2015.txt”, header = TRUE, sep = “”, row.names=1)

dim(genus) genus[1:5,1:5]

cooccur.genus <- cooccur(mat=genus,type=“spp\_site”, thresh=TRUE, spp\_names=TRUE)

# different ways for seeing the results - Genus Level L6

summary(cooccur.genus) pair.attributes(cooccur.genus) pair.profile(cooccur.genus) plot(cooccur.genus) print(cooccur.genus) prob.table(cooccur.genus)

# Alpha diversity of timeseries, for the 4 mice that were followed over 200 days, using a rarefied (at 5400 reads) table made in QIIME (so that we use the same rarefaction)

# First, create a new phyloseq object using the rarefied table made in Qiime

# Adding the treefile is not needed and might remove some OTUs, so I merged only rarefied OTU table and mapping file here.

OTUtableRar <- import\_biom(“Yana\_rarOTUtable\_SelectionPlusReps\_Rarefied5400\_13June2015.biom”) mapfile <- import\_qiime(mapfilename=“Yana\_Selection190samples\_13reps\_Mapping\_June2015.txt”)

psRar <- merge\_phyloseq(OTUtableRar, mapfile) print(psRar)

# Second, make a subset selection of the long timeseries (4 mice, all their timepoints) and check that they all still have 5400 reads per sample

tsRar <- subset\_samples(psRar, LongTimeSeries==“1”) print(tsRar) sample\_sums(tsRar)

# Now, calculate and plot Alpha diversity of the long timeseries, split per mouse (n=4), rarefied set

alpha\_tsRar <- plot\_richness(tsRar, x=“Days”, color=“Individual”, measures=c(“Observed”, “Simpson”, “Shannon”), title=“TimeSeries AlphaDivRar5400”) + geom\_point(size = 4, alpha = 0.8) + geom\_line(linetype=1) alpha\_tsRar

# Exporting the alpha diversity values for each timepoint, rarefied

alpha\_tsRar\_est <- estimate\_richness(tsRar, split=TRUE, measures=NULL) alpha\_tsRar\_est[1:10,] write.table(alpha\_tsRar\_est,“AlphaDiv\_TimeSeries\_June2015\_rarefied5400.txt”, sep=“”, quote=F, col.names=NA)

# Redo the same alpha diversity calculations, but now for the first 1-4 time points for ALL 46 mice, so only looking at the first 2 weeks or so

# First make a subset based on column “FourTPs” in mapping file (excludes long timeseries)

fRar <- subset\_samples(psRar, FourTPs==“1”) print(fRar) sample\_sums(fRar)

# Second, calculate and plot Alpha diversity of the long timeseries, split per mouse (n=4), rarefied set

alpha\_fRar <- plot\_richness(fRar, x=“Days”, color=“Individual”, measures=c(“Observed”, “Simpson”, “Shannon”), title=“First 4 timepoints AlphaDivRar5400”) + geom\_point(size = 4, alpha = 0.8) + geom\_line(linetype=1) alpha\_fRar

# Exporting the alpha diversity values for each timepoint, rarefied

alpha\_fRar\_est <- estimate\_richness(fRar, split=TRUE, measures=NULL) alpha\_fRar\_est[1:10,] write.table(alpha\_fRar\_est,“AlphaDiv\_First4Timepoints\_June2015\_rarefied5400.txt”, sep=“”, quote=F, col.names=NA)
